# Detection of enterotoxigenic *Bacteroides fragilis* in patients with ulcerative colitis

**DOI:** 10.1186/s13099-017-0202-0

**Published:** 2017-09-15

**Authors:** Samin Zamani, Sonia Hesam Shariati, Mohammad Reza Zali, Hamid Asadzadeh Aghdaei, Akram Sarabi Asiabar, Saied Bokaie, Bizhan Nomanpour, Leonardo Antonio Sechi, Mohammad Mehdi Feizabadi

**Affiliations:** 1 Department of Microbiology, School of Medicine, Tehran University of Medical Sciences, Tehran, Iran; 2 Department of Microbiology, School of Medicine, Golestan University of Medical Sciences, Gorgan, Iran; 3 Basic and Molecular Epidemiology of Gastrointestinal Disorders Research Center, Research Institute for Gastroenterology and Liver Diseases, Shahid Beheshti University of Medical Sciences, Tehran, Iran; 4 Division of Epidemiology & Zoonoses, Department of Food Hygiene and Quality Control, Faculty of Veterinary Medicine, University of Tehran, Tehran, Iran; 5 Department of Microbiology, School of Medicine, Kermanshah University of Medical Sciences, Kermanshah, Iran; 6 Department of Biomedical Sciences, University of Sassari, Viale San Pietro 43b, 07100 Sassari, Italy; 7 Thoracic Research Center, Imam Khomeini Hospital, Tehran, Iran

**Keywords:** Enterotoxigenic *Bacteroides fragilis*, ETBF, Ulcerative colitis, IBD, *bft*, Real time PCR

## Abstract

**Purpose:**

Ulcerative colitis (UC) as a type of inflammatory bowel disease (IBD), presumed to occur as a consequence of increased immune responses to intestinal microbiota in genetically susceptible individuals. Enterotoxigenic *Bacteroides fragilis* (ETBF) strains are important intestinal bacteria that can be involved in IBD. The aim of this study was to design a quantitative assay for detection of *B. fragilis* and ETBF and also to find their association with UC.

**Methods:**

Ninety-five biopsies were collected from patients with UC (n = 35) and with no IBD (nIBD, n = 60). All the specimens were cultured in Bacteroides bile esculin agar medium. Specific primers and probes were designed for quantitative real-time PCR (QRT-PCR) based on 16S rRNA and *bft* genes sequences of ETBF.

**Results:**

The *bft* genes were detected in 51.4% of UC samples and 1.6% of nIBD samples, respectively. In UC patients, 37.1% of samples with diarrhea and 11.4% of samples without diarrhea, harbored the *bft* gene. Mean value of the number of ETBF with *bft* gene in UC and nIBD samples were 4.46 ×Ÿ 10^2^ and 1.96, respectively. Likewise these result for 16S rRNA gene in UC and nIBD samples were 2.0 × 10^3^ and 8.4 × 10^3^, respectively.

**Conclusions:**

There was no significant association between presence and numbers of 16S rRNA gene of *B. fragilis* and UC. ETBF was detected more in UC specimens and biopsies of UC patients with diarrhea than in the control group. These data demonstrated that ETBF is associated with development of UC and as a causative agent for the development of diarrhea in these patients.

## Background

Ulcerative colitis (UC) is a type of human inflammatory bowel disease (IBD) caused by improper activation of the intestinal mucosa as a consequence of combination of interactive factors such as genetic, immunological and environmental [1, 2]. The composition of intestinal microbiota may be a factor in the initiating or perpetuating UC. The intestinal microbiota affects some important functions such as the activity of epithelial cells, immune regulation and intestinal mucosal inflammation [2]. Moreover, increasing evidence implicates a role of altered intestinal microbiota in the pathogenesis of several gastrointestinal (GI) disorders such as UC [3, 4]. The role of the intestinal microbiota in the etiology of these inflammatory diseases has yet to be defined, but a large number of investigations showed that UC is a consequence of the immune cell response to the constant antigenic stimulation of intestinal microbiota and the corresponding metabolites [5, 6]. *Helicobacter* spp., *Mycobacterium avium* subsp. *paratuberculosis*, *Listeria monocytogenes* and several other bacterial pathogens have also often been related to UC [7, 8].

In addition, the protective effects of antibiotics that target anaerobic gut bacteria have been observed in different type of UC, suggesting the possible role of the anaerobic bacteria in the pathogenesis of such inflammatory disease [9, 10].

Numerous studies have specified that the most bacteria in the human gut belong to two phyla including Gram negative Bacteroidetes and Gram positive Firmicutes [11]. Bacteroidetes are more transcriptionally active compared to Firmicutes at the mucosal surfaces [12]. The composition of intestinal microbiota in the fecal and mucosal surface is different. Several studies have been published in regards to mucosa-associated bacterial flora, due to significance of mucosal surface in UC [3, 13, 14]. *Bacteroides fragilis* is one of the intestinal microbiota species, present in the normal colon of most adults. Strains of enterotoxigenic *B. fragilis* (ETBF) secrete a 20-kDa proinflammatory zinc-dependent metalloprotease, stimulating the high expression of host interleukin-17. Furthermore, the permeability of the gut epithelial cells can be enhanced as a consequence of *B. fragilis* enterotoxin (BFT), resulting in the intensification of internalization of different enteric bacteria. This action is considered as one of the important routes for delivering an antigen to the active immune cells and consequently initiating a tissue invasion [15]. In animals and humans, ETBF functions can lead to severe inflammation in IBD, resulting in Crohn’s disease (CD) and UC as well as diarrhea and colorectal cancer (CRC) [3, 16–20].

In UC patients, not enough data are available for quantitative detection of ETBF using biopsies. In this study, in order to determine the potential association between particular bacterial populations and intestinal inflammation in UC, the mucosal tissue of UC patients and nIBD controls were examined for the presence of ETBF using quantitative real time PCR (QRT-PCR). The data from QRT-PCR were also used to determine the association of total mucosa-associated bacterial flora with disease state in UC patients.

## Methods

### Patients and tissue specimens

In this study, in total 95 biopsies were collected from Iranian UC patients (n = 35) as well as patients with non-inflammatory IBD (nIBD) (n = 60), using colonoscopy. Patients were referred to Taleghani hospital and Behboud clinic between March 2015 and May 2016. Ethics Committee of Tehran University of Medical Sciences approved the study protocol (IR.TUMS.REC.1395.2655). The informed consent was obtained in all cases. All UC patients were diagnosed based on clinical symptoms as well as endoscopic, histologic and radiographic standards and shown typical features such as active colitis with ulceration and mucosal inflammation with special distribution [21]. All the UC biopsy samples were collected from involved regions of terminal ileum and colon, found in colonoscopy. All of the patients presented in this study were newly diagnosed with UC and have not taken antibiotics or probiotics for the last 3 months. The controls were chosen from individuals with non-inflammatory IBD (nIBD), having a surgery because of abdominal pain or an alteration in bowel movement due to nIBD conditions. None of the individuals participating in this study took any antibiotics or probiotics for the last 3 months. Patients with any clinical and/or histopathological diagnosis of IBD were excluded from the study and the biopsies were obtained in the same way as did for patients with UC. All the samples were placed in sterile plastic containing thioglycollate medium (Merck, Germany) and transported in anaerobic condition to the laboratory for immediate processing.

### Bacterial culture of gut specimens

All biopsies were homogenized and subsequently cultured in anaerobic agar plates containing Bacteroides bile esculin agar (BBE) (Himedia Laboratories Pvt. Ltd, India). The plates incubated in an anaerobic chamber at 37 °C for 3 days. The confirmation of *B. fragilis* was done using PCR method.

### DNA extraction

DNA extraction was performed on biopsies using RTP^®^ Mycobacteria Kit (Invitek, Berlin, Germany) and preserved at −20 °C for further analysis with Real Time assays.

### Design primers and probes

The sequences of the *bft* gene and 16S rRNA gene were retrieved from Genebank. The primers and probes were designed using primer 3 plus based on sequences of conserved region of the genes (http://www.bioinformatics.nl/cgi-bin/primer3plus/primer3plus.cgi). The specificity of the primers and probes sequences was determined by comparison of all available sequences, using BLAST database search program (http://www.ncbi.nlm.nih.gov/BLAST).

All the primers and probes designed in this study are shown in Table 1.

**Table 1:** Sequence of the primers and probes

Gene name	Primer F Primer R (5′–3′)	Probe (5′–3′)	Product size
16S rRNA	TGGACTGCAACTGACACTGA GCCGCTTACTGTATATCGCA	FAM-TCCTGTTTGATACCCACACTTTCGAGC-BHQ1	115
*bft*	TGAAGTTAGTGCCCAGATGC CAGTAAAGCCTTCCAGTCC	FAM-AAGTGGCGACGCCAAAGAGG-BHQ1	150

### Real time PCR

Genomic DNA was extracted from ETBF reference strain and used as positive control. The optical density (OD) of extracted DNA was defined at 260 nano meter by means of a NanoDrop 1000 (Thermo Scientifi c, USA). To make the standard DNA for amplification, the number of molecules of the template per gram was calculated using the following formula: molecules of DNA = mass (in grams) Avogadro’s number/average molecular weight of a base × template length [22]. The standard curve for *bft* and 16S rRNA gene was assessed using each primers and probe sets with a tenfold serial dilution of *B. fragilis* DNA samples, corresponding to 10^1^–10^6^ Mean value/g of biopsies.

According to the standard curve and *y*-intercept, samples which did not display the fluorescent signal earlier than the Ct of 37 were considered as negative. Samples that produced fluorescence ≥10 were diluted. Likewise, at a concentration of more than 10^11^ cells, the curve does not follow linearity [22]. The efficacy of the real-time PCR was calculated by following formula: E = 10^(−1/slop)^ − 1 [22].

After optimization and qualification of standards curves, the dilution series was included in each amplification run.

Real-time PCR assays were performed using LinGene K Real Time PCR apparatus (Bioer, Hangzhou, P.R. China). All assays were performed in a total volume of 25 µl consisting of 2× TaqMan universal master mix (Applied Biosystems, Foster City, CA), 0.2 mM each primer, 0.1 mM TaqMan probe, and 2 ng DNA in double distilled water. The thermocycler program for *bft* gene was 95 °C for 10 min for initial denaturation, followed by 40 cycles of a two-stage temperature profile of 95 °C for 10 s and 62 °C for 1 min. PCR condition for 16S rRNA gene was 95 °C for 10 min, followed by 40 cycles of 95 °C for 20 s and 60 °C for 1 min. To control the quality all the tests were performed in duplicate and mean was reported. The negative control was a PCR TaqMan master mix without DNA.

To check the specificity of the PCR and expected size of the product, the primers were applied in a conventional PCR and the amplicons were run on agarose gel. Also Specificity of positive amplified fragments was confirmed by automated sequencing.

### Statistical analysis

Chi square test, Fisher’s exact test and Mann–Whitney Test were applied to determine the statistical significance of the data obtained for the presence of ETBF. P value <0.05 was considered statistically significant. Mean values ± std. Error of mean (SEM) were computed for *B. fragilis* and ETBF. Statistical analysis of the data was conducted using the SPSS 13.0 software program.

## Results

Thirty-five samples were taken from UC patients (14 males and 21 females; mean age 36.71 years, range 16–75) and 60 samples were taken from nIBD (28 males and 32 females; mean age 36.9 years, range 16–75).

Among 35 UC samples and 60 nIBD, 16 (45.7%) and 31 (51.7%), respectively were culture positive for *B. fragilis* (Table 2) (*P* = 0.364).

**Table 2:** Data regarding *B. fragilis* bacterial culture

	Culture of		Total
	No	Yes	
nIBD			
Count	29	31	60
nIBD (%)	48.3	51.7	100.0
UC			
Count	19	16	35
UC (%)	54.3	45.7	100.0
Total			
Count	48	47	95
Total (%)	50.5	49.5	100.0

**Fig. 1:**
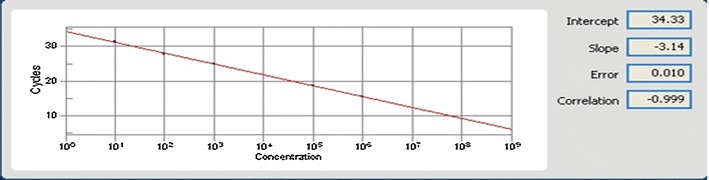
Analysis of data for Real time PCR. In standard curve, X and Y axis showed the concentration of ETBF and number of cycles respectively for control positive sample

According to standard curve dilutions of ETBF (control positive) DNA at 10^1^, 10^2^, 10^3^, 10^4^, 10^5^ and 10^6^ provided Ct values of 15.48 ± 0.2, 18.74 ± 0.2, 21.44 ± 0.3, 24.87 ± 0.4, 27.71 ± 0.4 and 31.46 ± 0.4, respectively. The efficacy of the real-time PCR was between 95% and 100% (Fig. 1). Furthermore, all standard dilutions and specimens had 1 band in the gel electrophoresis (data not shown). Also samples which did not display the fluorescent signal earlier than 37 cycles were considered negative.

The number of positive samples for 16S rRNA gene and *bft* gene, using real time PCR are presented in Table 3.

**Table 3:** Quantitative analysis of the 16S rRNA gene and *bft* genes from UC and nIBD biopsy samples

	No. of positive samples	
	*B. fragilis*	ETBF
nIBD		
Count	45	1
nIBD (%)	75	1.6
UC		
Count	24	18
UC (%)	68.5	51.4
P value	0.328	0.000

The results of quantitative analysis of real time PCR for 16S rRNA gene counted for *B. fragilis* and *bft* gene counted for ETBF were shown in Table 4.

**Table 4:** Quantitative analysis of the 16S rRNA gene and *bft* genes from UC and nIBD biopsy samples

	*B. fragilis*	ETBF
nIBD		
Mean	412.7	.03
N	60	60
SEM	199.9	.03
UC		
Mean	171.6	83.4
N	35	35
SEM	69.8	28.1
P value	0.7	0.0

*SEM* std. error of mean

In this study there were 60 non-IBD controls. Of whom 21% (13/60) had diarrhea. One (7%:1/13) was bft positive and 21% (47/60) didn’t had diarrhea none of them (0%:0/13) was bft positive. Differences between the percentage of bft positive in non-IBD controls with diarrhea compared to those without diarrhea wasn’t statistically significant (*P* = 0.21).

In the UC group 57% (20/35) had diarrhea of whom 65% (13/20) were bft positive. In the “UC” group 43% (15/35) had no diarrhea, of whom 26% (4/15) were bft positive Differences between the percentage of bft positive in the UC group with diarrhea compared to those without diarrhea was statistically significant (*P* = 0.04) (Table 5).

**Table 5:** ETBF in patients with and without diarrhea

	ETBF	
	Negative	Positive
UC		
Diarrhea (n)	7	13
No diarrhea (n)	11	4
Total (n)	35	
P value	0.04	
nIBD		
Diarrhea (n)	12	1
No diarrhea (n)	47	0
Total (n)	60	
P value	0.21	

The analysis of the results of sequencing approved the presence of *bft* and 16S rRNA gene. Also these result determined the presence of the *bft1* subtype in all the positive samples.

## Discussion

IBD is known as a heterogeneous disease consisting of two main types: UC and CD [2]. In the recent years the prevalence and incidence of UC have been increased in Iran [23, 24], following the same trend in other developing countries.

There is not enough data to describe precisely the causative agents for these intestinal disorders. In addition, the etiology of the disease is still unknown [2]. The influence of gut microbiota on human health has been widely debated and some studies recommended that alteration of gut flora might lead to different disorders [25]. In a study conducted by Lucke et al. the gut microbiota was analyzed in different UC patients and some pathogens responsible for initiating, developing and relapsing of the disease have been identified [3].

Microbial symbiosis is among the main environmental risk factor for development of UC. Previous studies have shown that ETBF could be a major factor in initiating or relapsing of UC [16]. No meaningful data exist regarding the role of ETBF in human disease. Some studies reported that ETBF could be detected in normal human gut microbiota as well as in infectious samples [26, 27]. There is emerging evidence that introduced the connection of ETBF-associated inflammatory diarrheal diseases with IBD and CRC [15, 17, 18, 28, 29]. Furthermore, some studies have been published recently, describing ETBF as a trigger for chronic Stat3/IL-17-driven colitis which is IL-17 dependent and induces Th17 colitis and tumorogenesis through the secretion of the metalloproteinase Bft and other associated factors [30, 31]. Also some data showed that ETBF may initiate both a systemic as well as a mucosal inflammatory response [17]. Since the chronic inflammation as a consequence of IBD is a major risk factor for development of CRC, thus determination of the factors associated with IBD might also be important in finding the etiology of the disease [32, 33].

There was no difference in the incidence of *B. fragilis* positive culture between UC and control patients (Table 2). In some studies, Bacteroidetes were present more in the mucosal biopsy of IBD patients including UC and CD compared to the controls, determined by culture-based methods [3, 34]. The gold standard method for detection of bacteria is culture-based but it requires a high number of viable cells and specifically for anaerobic bacterial culture, some limitations apply.

Therefore, different methods like real time PCR can be used for detection and quantification of these organisms. The rate of *B. fragilis* harboring *bft* gene was higher in UC population in comparison with nIBD, as found in previous studies [14, 18]. Although we found that 51.4% of UC samples harbored *bft* gene, an increased rate of ETBF, compared to the preceding reports [16]. Also quantitative real time PCR showed higher mean value of *bft* gene in biopsy of UC samples compared to nIBD. In both groups, no difference in the incidence of the *bft* gene could be found either between male or female. Moreover according to the result of sequencing, subtype of *bft* gene was *bft1* in all the positive samples. Further studies are needed to focus in other subtypes of this gene. Other studies found *bft*-*1* to be the predominant subtype [35, 36]. Further studies are needed to focus in other subtypes of this gene.

Since ETBF strains are associated with diarrheal diseases, their presence in UC samples with diarrhea symptoms was examined. Our data demonstrated that ETBF was detected significantly and more frequently in the samples of UC patients with diarrhea symptoms than in others without diarrhea and in control patients. So we may consider ETBF as one of the factor associated with diarrhea in UC patients. Several studies implicated that *B. fragilis* which harbored *bft* gene are more present in patients with diarrhea and IBD compared to control patients with no diarrhea [16, 20]. A recent study in Iran has considered ETBF as one of the causative agents of diarrhea in children less than 5 years old [37].

Also real time PCR showed less 16S rRNA gene in the UC samples compared with nIBD samples although this difference was not statistically significant. These data suggest that probably the strains that harbor *bft* gene, not *B. fragilis* by itself, could contribute to UC in this study. Some studies presented that Bacteroides can be exist more in IBD patients than normal controls in FISH and conventional culture methods that were commonly from European patients. But some investigations showed lower levels of Bacteroides in IBD patients compared to the controls that the samples were mostly obtained from Asian patients [38, 39]. This finding proposed Bacteroides levels may be attributed not only to the types of detection method but also to patient demographics and ethnic groups. Additionally, we didn’t analysis other species of Bacteroides and other virulence factor of it, so maybe in our population need to study them.

Based on the present data, polysaccharide A (PSA) from the capsule of *B. fragilis* will be a good potential target to be taken into consideration in the future study.

## Conclusions

In conclusion, considering the real time results for *bft1* gene, the high incidence of ETBF in mucosal biopsies of UC patients was statistically significant. Also our results suggest that ETBF through secretion of its pro-inflammatory toxin (BFT), is capable of initiation or development of diarrhea symptoms in the susceptible UC host. In order to further investigate the influence of ETBF in UC development, other environmental risk factors in the population should be determined. Data of this study show a decrease in the numbers of *B. fragilis* in the colonic mucosa of UC patients compared to control group, suggesting the potential involvement of other virulence factors in *B. fragilis* which has to be taken into consideration for further clarification of the issue.. Increasing the sample size and studying different population would be necessary to confirm this association. Based on the importance of IBD in the development of CRC, these data may pave the way for better understanding of the mechanism of UC in the corresponding population and help to find a proper tool in order to prevent the further development of bacteria.

## Abbreviations

IBD: inflammatory bowel disease
CD: Crohn’s disease
UC: ulcerative colitis
ETBF: enterotoxigenic *B. fragilis*
CRC: colorectal cancer
QRT-PCR: quantitative real time PCR

## References

[1] Ley RE, Peterson DA, Gordon JI (2006). Ecological and evolutionary forces shaping microbial diversity in the human intestine. Cell.

[2] Sartor RB (2010). Genetics and environmental interactions shape the intestinal microbiome to promote inflammatory bowel disease versus mucosal homeostasis. Gastroenterology.

[3] Lucke K, Miehlke S, Jacobs E, Schuppler M (2006). Prevalence of *Bacteroides* and *Prevotella* spp. in ulcerative colitis. J Med Microbiol.

[4] Cummings JH, Macfarlane GT, Macfarlane S (2003). Intestinal bacteria and ulcerative colitis. Curr Issues Intest Microbiol.

[5] Sartor RB (2006). Mechanisms of disease: pathogenesis of Crohn’s disease and ulcerative colitis. Nat Clin Pract Gastroenterol Hepatol.

[6] Khor B, Gardet A, Xavier RJ (2011). Genetics and pathogenesis of inflammatory bowel disease. Nature.

[7] Campieri M, Gionchetti P (2001). Bacteria as the cause of ulcerative colitis. Gut.

[8] Zamani S, Zali MR, Aghdaei HA, Sechi LA, Niegowska M, Caggiu E, Keshavarz R, Mosavari N, Feizabadi MM (2017). *Mycobacterium avium* subsp. paratuberculosis and associated risk factors for inflammatory bowel disease in Iranian patients. Gut Pathog.

[9] van Kruiningen HJ (1995). On the use of antibiotics in Crohn’s disease. J Clin Gastroenterol.

[10] Shen BB, Qian JM (2008). Intestinal flora and ulcerative colitis. Pract J Clin Med.

[11] Eckburg PBBE, Bernstein CN, Sargent M, Purdom EA, Relman DA (2005). Molecular analysis of the colonic mucosal microbiota in patients with Crohn’s disease. Science.

[12] Rehman A, Lepage P, Nolte A, Hellmig S, Schreiber S (2010). Transcriptional activity of the dominant gut mucosal microbiota in chronic inflammatory bowel disease patients. J Med Microbiol.

[13] Zoetendal EG, von Wright A, Vilpponen-Salmela T, Ben-Amor K, Akkermans AD (2002). Mucosa-associated bacteria in the human gastrointestinal tract are uniformly distributed along the colon and differ from the community recovered from feces. Appl Environ Microbiol.

[14] Gophna U, Sommerfeld K, Gophna S, Doolittle WF, van Zanten SJV (2006). Differences between tissue-associated intestinal microfloras of patients with Crohn’s disease and ulcerative colitis. J Clin Microbiol.

[15] Wells CL, van de Westerlo EM, Jechorek RP, Feltis BA, Wilkins TD, Erlandsen SL (1996). *Bacteroides fragilis* enterotoxin modulates epithelial permeability and bacterial internalization by HT-29 enterocytes. Gastroenterology.

[16] Prindiville TP, Sheikh RA, Cohen SH, Tang YJ, Cantrell MC, Silva J (2000). *Bacteroides fragilis* enterotoxin gene sequences in patients with inflammatory bowel disease. Emerg Infect Dis.

[17] Rabizadeh S, Rhee KJ, Wu S, Huso D, Gan CM, Golub JE, Sears CL (2007). Enterotoxigenic *Bacteroides fragilis*: a potential instigator of colitis. Inflamm Bowel Dis.

[18] Sack RB, Myers LL, Almeido-Hill J, Shoop DS, Bradbury WC, Reid R, Santosham M (1992). Enterotoxigenic *Bacteroides fragilis*: epidemiologic studies of its role as a human diarrhoeal pathogen. J Diarrhoeal Dis Res.

[19] Pathela P, Hasan KZ, Roy E, Alam K, Huq F, Siddique AK, Sack RB (2005). Enterotoxigenic *Bacteroides fragilis*-associated diarrhea in children 0–2 years of age in rural Bangladesh. J Infect Dis.

[20] Basset C, Holton J, Bazeos A, Vaira D, Bloom S (2004). Are *Helicobacter* species and enterotoxigenic *Bacteroides fragilis* involved in inflammatory bowel disease?. Dig Dis Sci.

[21] Dignass A, Eliakim R, Magro F, Maaser C, Chowers Y, Geboes K (2012). Second European evidence-based consensus on the diagnosis and management of ulcerative colitis part 1: definitions and diagnosis. J Crohns Colitis.

[22] Adams PS, Dorak MT (2006). Data analysis and reporting. Real-time PCR.

[23] Gismera CS, Aladren BS (2008). Inflammatory bowel diseases: a disease(s) of modern times? Is incidence still increasing?. World J Gastroenterol.

[24] Daryani NE, Bashashati M, Aram S, Hashtroudi AA, Shakiba M, Sayyah A (2006). Pattern of relapses in Iranian patients with ulcerative colitis. A prospective study. J Gastrointest Liver Dis.

[25] Musso G, Gambino R, Cassander M (2011). Interactions between gut microbiota and host metabolism predisposing to obesity and diabetes. Annu Rev Med.

[26] Cáceres M, Zhang G, Weintraub A, Nord CE (2000). Prevalence and antimicrobial susceptibility of enterotoxigenic *Bacteroides fragilis* in children with diarrhea in Nicaragua. Anaerobe.

[27] Scotto d’Abusco AS, M Del Grosso and M Pantosti. Characterization of the enterotoxin gene of *Bacteroides fragilis* strains from different human sources, abstr. 3.106, p. 56. In: Abstracts of the 2nd world congress on anaerobic bacteria and infections. Boston: International Society of Anaerobic Bacteria; 1998.

[28] Sears CL, Islam S, Saha A, Arjumand M, Alam NH, Faruque AS (2008). Association of enterotoxigenic *Bacteroides fragilis* infection with inflammatory diarrhea. Clin Infect Dis.

[29] Boleij A, Hechenbleikner EM, Goodwin AC, Badani R, Stein EM, Lazarev MG (2015). The *Bacteroides fragilis* toxin gene is prevalent in the colon mucosa of colorectal cancer patients. Clin Infect Dis.

[30] Rhee KJ, Wu S, Wu X, Huso DL, Karim B, Franco AA (2009). Induction of persistent colitis by a human commensal, enterotoxigenic *Bacteroides fragilis*, in wild-type C57BL/6 mice. Infect Immun.

[31] Sears CL, Geis AL, Housseau F (2014). *Bacteroides fragilis* subverts mucosal biology: from symbiont to colon carcinogenesis. J Clin Investig.

[32] Herrinton LJ, Liu L, Levin TR, Allison JE, Lewis JD, Velayos F (2012). Incidence and mortality of colorectal adenocarcinoma in persons with inflammatory bowel disease from 1998 to 2010. Gastroenterology.

[33] Gillen CD, Walmsley RS, Prior P, Andrews HA, Allan RN (1994). Ulcerative colitis and Crohn’s disease: a comparison of the colorectal cancer risk in extensive colitis. Gut.

[34] Hartley MG, Hudson MJ, Swarbrick ET, Hill MJ, Gent AE, Hellier MD, Grace RH (1992). The rectal mucosa-associated microflora in patients with ulcerative colitis. J Med Microbiol.

[35] Kato N, Liu CX, Kato H, Watanabe K, Tanaka Y, Yamamoto T, Suzuki K, Ueno K (2000). A new subtype of the metalloprotease toxin gene and the incidence of the three bft subtypes among *Bacteroides fragilis* isolates in Japan. FEMS Microbiol Lett.

[36] Avila-Campos MJ, Liu C, Song Y, Rowlinson MC, Finegold SM (2007). Determination of bft gene subtypes in *Bacteroides fragilis* clinical isolates. J Clin Microbiol.

[37] Akhi MT, Seifi SJ, Asgharzadeh M, Rezaee MA, Oskuei SA, Pirzadeh T, Memar MY, Alizadeh N, Sofla HS. Role of Enterotoxigenic Bacteroides fragilis in Children Less Than 5 Years of Age With Diarrhea in Tabriz, Iran. Jundishapur journal of microbiology. 2016;9(6):e32163. 10.5812/jjm.32163 PMC5013289 27635209

[38] Zhou Y, Zhi F (2016). Lower level of bacteroides in the gut microbiota is associated with inflammatory bowel disease: a meta-analysis. Biomed Res Int.

[39] Takaishi H, Matsuki T, Nakazawa A, Takada T, Kado S, Asahara T, Kamada N, Sakuraba A, Yajima T, Higuchi H, Inoue N (2008). Imbalance in intestinal microflora constitution could be involved in the pathogenesis of inflammatory bowel disease. Int J Med Microbiol.

